# An inherited *FGFR2* mutation increased osteogenesis gene expression and result in Crouzon syndrome

**DOI:** 10.1186/s12881-018-0607-8

**Published:** 2018-05-30

**Authors:** Jiayan Fan, Yinwei Li, Renbing Jia, Xianqun Fan

**Affiliations:** 1 Department of Ophthalmology, Ninth People’s Hospital, Shanghai JiaoTong University School of Medicine, Shanghai, People’s Republic of China; 2 Shanghai Key Laboratory of Orbital Diseases and Ocular Oncology, Shanghai, China

**Keywords:** Crouzon syndrome, *FGFR2*, Orbital volume, Osteoblast genes

## Abstract

**Background:**

*FGFR2* encodes a fibroblast growth factor receptor whose mutations are responsible for the Crouzon syndrome, involving craniosynostosis and facial dysostosis with shallow orbits. However, few reports are available quantifying the orbital volume of Crouzon syndrome and there was little direct evidence to show *FGFR2* mutation actually influencing orbital morphology.

**Methods:**

Ten Crouzon syndrome patients underwent a standard ophthalmologic assessment. Morphology study was carried out based on 3-dimensional computed tomography scan to calculate orbital volume. Genomic DNA was extracted from peripheral blood leukocytes of the patients and genomic screening of *FGFR2.* A three-dimensional computer model was used to analyse the structural positioning of the mutation site that was predicted possible impact on functional of FGFR2 protein. Real-time PCR was performed to analyse the expression of bone maker gene.

**Results:**

We describe a *FGFR2* mutation (p.G338R, c.1012G > C) in a Chinese family with Crouzon syndrome. Computational analysis showed the mutate protein obviously changes in the local spatial structure compared with wild-type FGFR2. The expression of osteocalcin and alkaline phosphatase two osteoblast specific genes significantly increased in orbital bone directly from patient compared to normal individual, which may lead to facial dysostosis. This is compatible with the shallow and round orbits in our Crouzon syndrome patient.

**Conclusions:**

Our study further identified G338R FGFR2 mutation (c1012G > C) lead to inherited Crouzon syndrome. Thus, early intervention, both medically and surgically, as well as disciplined by a multiple interdisciplinary teams are crucial to the management of this disorder.

## Background

Crouzon syndrome is characterized by craniosynostosis and facial dysostosis with an incidence of 16.5/1,000,000, accounting for 4.8% of all craniosynostoses [1]. In ophthalmology, ocular involvement can include variations ranging from exophthalmos and divergent strabismus to ocular hypertelorism [2]. The proptosis which can in turn put patients at increased for conjunctivitis, is secondary to shallow orbits. Currently, Crouzon syndrome is commonly diagnosed when patients present with obviously facial structure abnormalities and growth patterns. However, exophthalmos was happening largely before these presentations as exophthalmos was visualized on fetal ultrasound at 35 weeks for orbital growth retardation [3]. Unfortunately, few reports are available quantifying the orbital volume of Crouzon syndrome.

Molecular findings prove it is a rare autosomal dominant disease. Mutations in *FGFR2*, a gene encoding fibroblast growth factor receptor type 2 (*FGFR2*) located at 10q26 have been identified in both syndromic and non-syndromiccraniosynostosis [4–7]. It consists of three extracellular immunoglobulainlike (Ig) domains, a single transmembrane segment, and a split tyrosine kinase domain [8]. Although a number of mutations of *FGFR2* have been well characterized to date and a few mutations are identified affecting cell proliferation such as osteoblasts and chondrocytes in vitro, [9–11] however, to our knowledge, there is no literature reported the expression of osteoblasts and chondrocytes in vivo, especially in orbital bone from Crouzon syndrome patient.

In this manuscript, we have attempted to identify the gain-of-function role of G338R FGFR2 (c.1012G > C) potentially related to shallow orbits even premature suture closure in Crouzon syndrome patient. We found OC (osteocalcin) and ALP (alkaline phosphatase) two selected bone marker genes were significantly increased in the patient’s orbital bone which indicate G338R FGFR2 may affect orbital bone development.

## Methods

### Ethics statement and clinic examination

The research was carried out according to the principles of the Declaration of Helsinki; informed consent was obtained and Shanghai Ninth People’s Hospital Ethics Committee approved the study. The patient photos, which are contained in this article, were taken by a hospital-based photographer at Shanghai Ninth People’s Hospital, Shanghai Jiao Tong University School of Medicine. Permission to use these photos in this report has been obtained from all the subjects including the parents of individual V2 and IV-2 (minor’s parent: legal guardian) who participated in this study.

We present 10 Crouzon syndrome patients from one Chinese family with 5 generation. All patients underwent a standard ophthalmologic assessment. Three-dimensional orbital computed tomography (CT) scan examination was used for bony defects. All CT scans were obtained with a spiral CT scanner (GE LightSpeed 16, Milwaukee, WI). The scans included the range from the maxilla to the skull. Continuous high-resolution scanning with a slice detection of 1.25*16 mm, reconstructed thickness of 1.25 mm, and slice increment of 1.25 mm was performed. The reconstructed field of view was 23 cm, and the reconstruction matrix was 512*512. All CT images were loaded in the DICOM format. Mimics v18.0 software (Materialise, Leuven, Belgium) was used for further calculations and analyses. After the DICOM data were imported into Mimics, the coordinate system was redefined to ensure that all measurements were completed in the same environment. The new coordinate system was defined according to the protocols of the “Online Reslice” and “Reslice Project” functions. The axial plane was defined as a plane parallel to the Frankfurt plane, passing through the inferior margin of the left orbit and the upper margin of each of the external auditory meatuses. The sagittal plane was defined as the median plane passing through the midpoint of the nasion, sellaturcica, and foramen magnum. The oblique sagittal plane was defined as the plane passing through the midpoint of the optic canal orifice and the ipsilateral orbital aperture and was perpendicular to the axial plane. The coronal datum plane was defined as the coronal plane passing through the most posterior point of the lateral orbital rim. The orbital width, orbital height, roof length, floor length, medial wall length, lateral wall length, ocular protrusion, bony orbital volume (BOV), midinterorbital distance and lateral orbital wall angle parameters were calculated according to the protocol in the previous study [12].

### FGFR2 mutation screening

Genomic DNA was extracted from peripheral blood leukocytes of the patients and 100 randomly selected healthy volunteers using the Automatic Nucleic Acid Isolation System (QuickGene-610 L; Fujifilm Life Science, Tokyo, Japan). PCR for the target sequence of *FGFR2* was performed, the products purified and sequenced as previously described [13, 14]. A three dimensional computer model was used to analyse the structural positioning of FGFR2-G338A mutation site. The immunoglobulin (Ig)-like domain 3 of FGFR2 protein was modeled with SWISS-MODEL (template PDB code, 1iil) as we have previously described [15, 16].

### Real-time00205PCR for osteocalcin (OC) and alkaline phosphatase (ALP)

Orbital bones obtained from Crouzon syndrome patient and orbital fracture patient on patient’s consent. After impurities removing, bone samples were soaked in lquid nitrogen and grined into fine particles or a powder. Total RNA was extracted using TRI-REAGENT (Invitro- gen, USA) according to the manufacturer’s instructions, and cDNA was synthesized using the PrimeScript RT re-agent kit (Takara, Japan) as we have previously described [17]. The housekeeping gene GAPDH was used as an endogenous control. The PCR cycle parameters for OC and ALP expression were as follows: 33 cycles of denaturation at 95 °C for 30 s, optimal annealing temperature for 30 s, extension at 72°Cfor30sandafinalextensionat72°Cfor5min.

All of the experiments were performed in triplicate, and the data were expressed as the mean ± standard deviation (SD). The comparative threshold cycle method was applied in the quantitative real-time RT-PCR assay according to the ΔΔ threshold cycle method.

## Results

### Patients and clinical characteristics

**Fig. 1:**
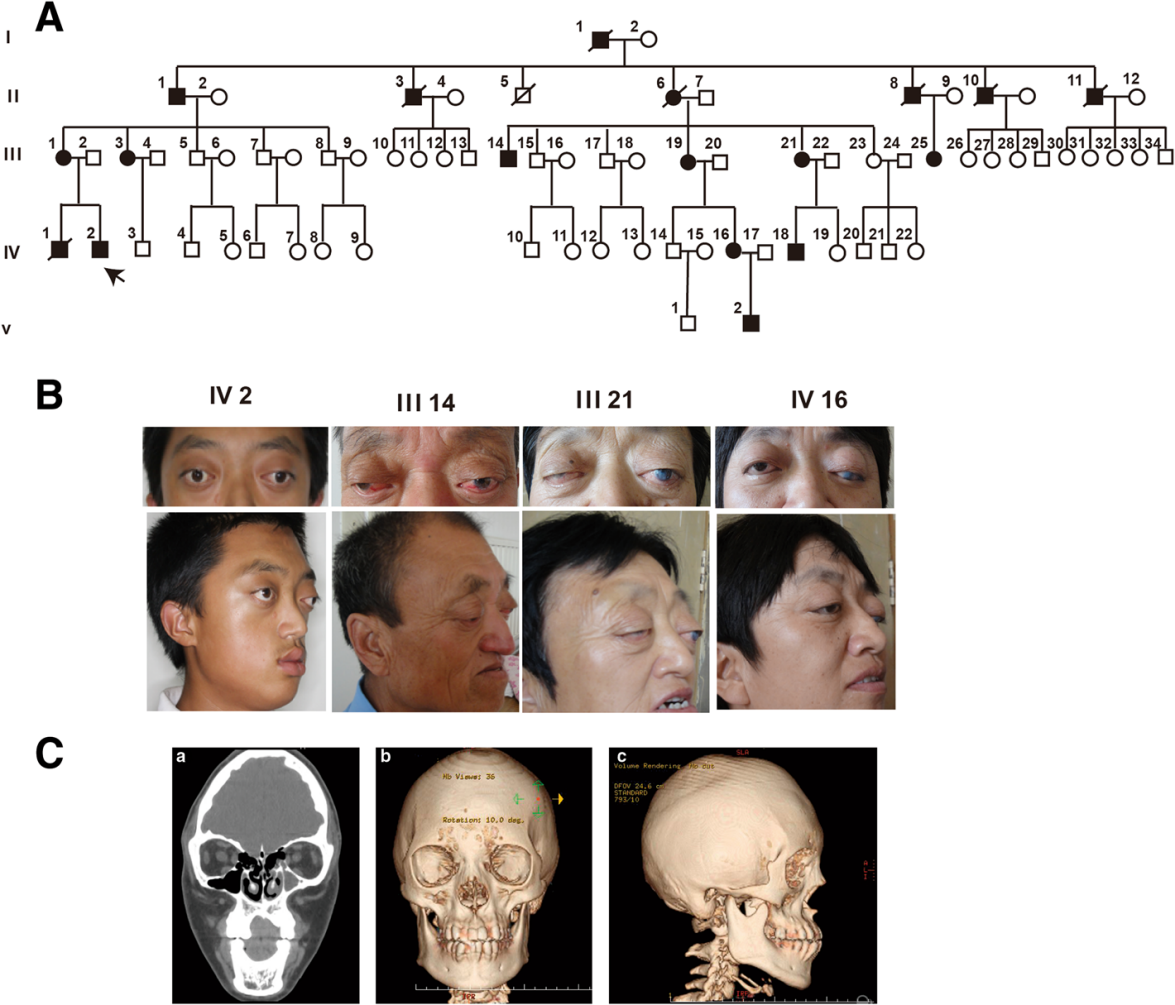
Crouzon syndrome patients and morphology measurement of orbit. **a** Five-generation Crouzon syndrome pedigree. Affected individuals are indicated by filled symbols, the proband is marked with an upward arrow. **b** Facial photographs of the Crouzon syndrome patients. **c** Morphology measurement of orbit. (Ca) Three-dimensional reconstruction of skull of the proband IV-2 (Cb) Morphology measurement of orbit(a. midinterorbital distance, b.lateral orbital wall angle) (Cc) Morphology measurement of orbit(a. roof length, b. medial wall length, c. floor length, d. lateral wall length, e. orbital width, f. orbital height)

We present 10 Crouzon syndrome patients from one Chinese family with 5 generation (Fig. 1a). These patients had shallow orbits and ocular proptosis, accompanied by craniosynostosis, mid-face hypoplasia, a curved, beaklike nose, but clinically normal hands and feet. They had normal vision since early childhood, just displaying a surprised look, but their vision was getting worse and worse as they aged. The visual acuity of individual III-14 (unmarried, 64 years old) was FC /1 m (figure count, OU). The quality of his life is at stake for serious exposure keratitis and obstinate conjunctivitis. And even more severe visual impairment, because of chronic corneal scarring result from hypophasis (Fig. 1b, III21and IV16), the visual acuity of III21 and IV16 was LP (light perception, OU of III21, OS of IV16).

Individual IV-2 (Fig. 1b, panel 1) the probands was born after a normal pregnancy and was referred for craniofacial assessment at 14 years of age because prominent eyes. He had a Crouzon syndrome brother (IV-1) died around 8 months old. The visual acuity of the IV-2 patient was 0.4 (OD) and 0.1 (OS). The refractive error was − 2.25D, with − 1.00 astigmatism (OD) and + 0.75D, with − 3.00 astigmatism (OS). Vertical interpalpebral fissure height (IPFH) OD 11 mm, OS 7 mm; horizontal palpebral fissure length (HPFL) OD 20 mm, OS 19 mm; levator function OD 5 mm, OS 8 mm; The prominence of the IV-2 patient was 20 mm (OD) and 18 mm (OS). In addition aural atresia was detected. No abnormalities were detected in the lenses, retinas, choroids, or optic nerves.

### Orbital morphology

To measurement the orbital volume, we carried out the morphology study. The data showed the orbital volume of Crouzon syndrome patient was much more shallower and rounder than normal population (Table 1). As the data show the shape of the orbital aperture was more round and bigger than the normal orbits, for both the orbital height and width were larger than what we have previously measured from normal male Chinese adults [12] for the bony orbit have almost reached the adult level in the 14-year old patient [18]. The patient orbits were quite shallow as all length parameters from the orbital aperture to the optic nerve canal orifice were smaller than the normal data, especially the medial wall length (OD 39.97 mm, OS 38.00 mm, normal 46.32 ± 2.67 mm) and the floor length (OD 37.38 mm, OS 34.66 mm, normal 47.93 ± 2.68 mm), which was consistent with the dysplasia of the maxilla,finally resulted in a small orbital cavity (OD 17.15 ml, OS 16.31 ml, normal 26.04 ± 2.60 ml).However,the patient ocular volume was 5.76 ml (OD) and 5.96 ml (OS)which was normal along with the shallow orbits finally caused the “uninhabitable habitation”, then exophthalmos was presented.

**Table 1:** Quantitative morphometry of the orbit in Crouzon syndrome patient and normal population [12]

	orbital width	orbital height	roof length	floor length	medial wall length	lateral wall length	ocular protrusion	BOV	Angle	Midwinter orbital distance
	OD/OS	OD/OS	OD/OS	OD/OS	OD/OS	OD/OS	OD/OS	OD/OS	OD/OS	
	mm	mm	mm	mm	mm	mm	mm	ml	degrees	mm
Patient	44.94/ 43.60	39.16/38.60	49.66/ 48.09	37.38/34.66	39.97/38.00	44.65/42.62	24.59/23.85	17.15/16.31	56.52°/55.94°	40.01
Normal	33.35 ± 1.44	40.02 ± 1.63	52.93 ± 2.89	47.93 ± 2.68	46.32 ± 2.67	48.38 ± 2.50	16.97 ± 2.87	26.04 ± 2.60	42°	30

Normal: 64 subjects who diagnose conditions other than craniofacial or orbital deformation

### G338R mutation was identified in *FGFR2* (Ig)-like domain

**Fig. 2:**
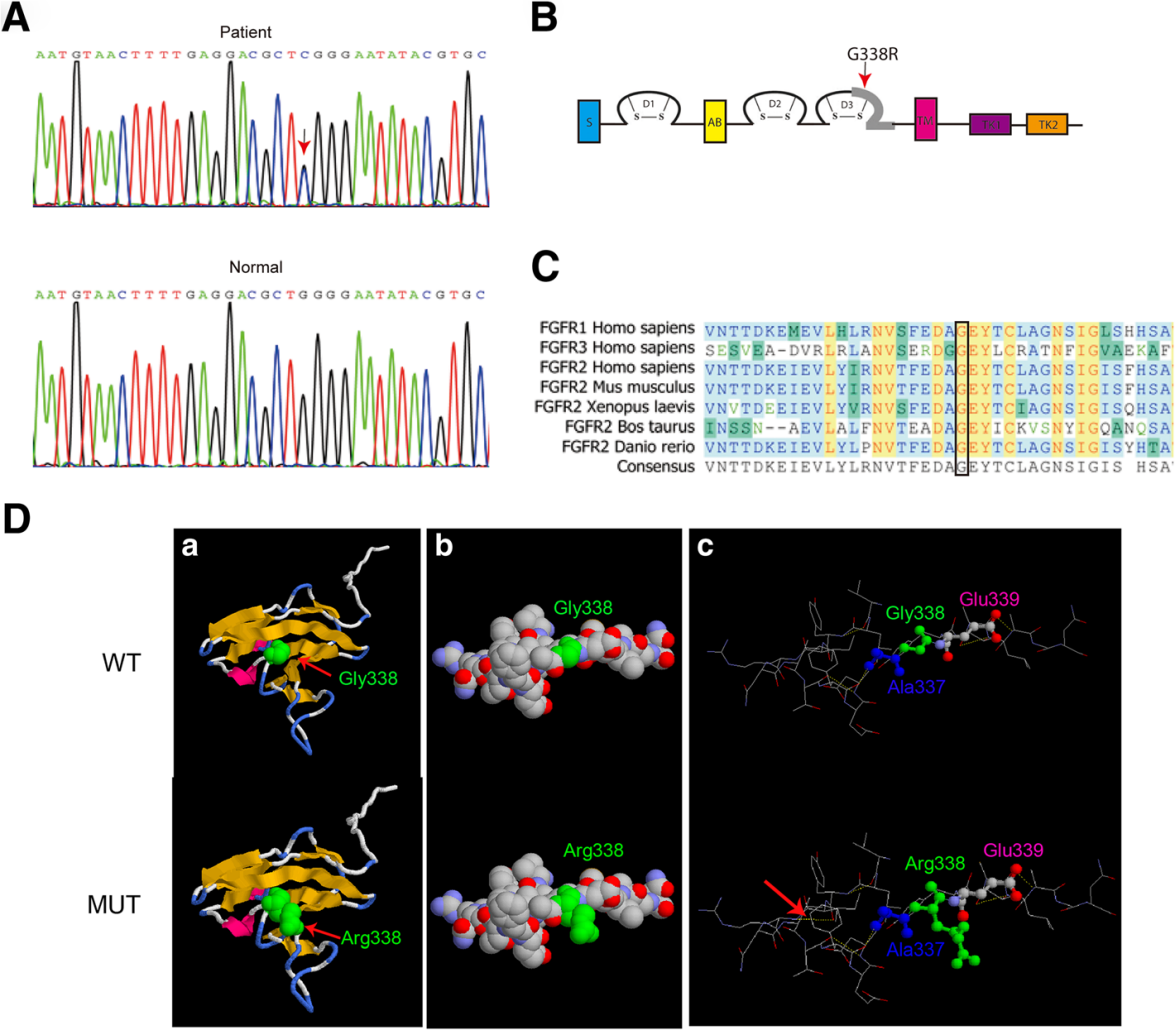
A heterozygous missense mutation was identified in FGFR2. **a** A heterozygous missense mutationin exon 10 of the *FGFR2* was identified in Crouzon syndromepatients which was absent in normal population. **b** Mapping of pathogenic FGFR2 mutations. **c** Amino acid sequence of FGFR2 around G338R in species of the vertebrates and FGFR family, a highly conserved segment of the FGFR2 protein. **d** Molecular modelling of the immunoglobulin (Ig)-like domain 3 of the wild-type and mutant-typep.G338RFGFR2

Sequencing of the complete coding sequence of the *FGFR2* gene derived from genomic DNA revealed a heterozygous mutation (p.G338R, c. 1012G > C) in exon 10 of the human FGFR2 (Fig. 2a), causing the replacement of glycine (Gly) by arginine (Arg) at amino acid position 338, a highly conserved segment of the FGFR2 protein (Fig. 2c). This mutation is located in the immunoglobulin (Ig)-like domain 3 (Fig. 2b). Moreover, the missense mutation was not found in 100 normal people, including III-5 and III-7 from the Crouzon syndrome family who were normal subjects (Fig. 2a).

To study the structural changes in the mutant-type FGFR2, three-dimensional structural model was used to analyse the position of missense mutation. Compared with wild-type FGFR2 (Fig. 2d, a-c), obviously changes were detected in the local spatial structure of the mutant-type FGFR2 (Fig. 2d, d-f) carrying the amino acid substitution in the (Ig)-like domain 3. Notably, in the wild-type FGFR2 there was no hydrogen bond between Tyr328 and Arg330 (Fig. 2d, c). However, in the mutant-type FGFR2one hydrogen bond was formed between Tyr328 and Arg330 that was near the Gly338 amino acid residue (Fig. 2d, f), which may reduced the stability of protein.

### OC and ALP mRNA level was increased in patient orbital bone

**Fig. 3:**
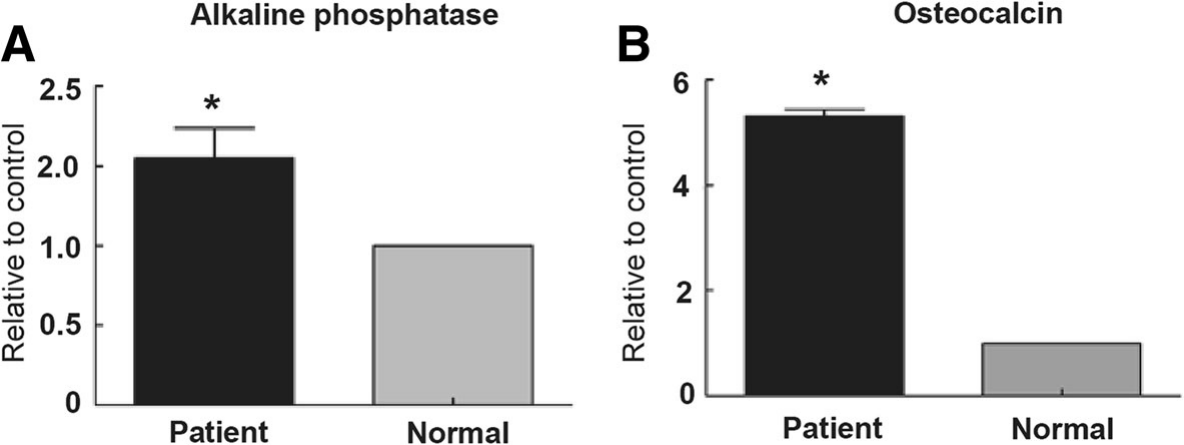
Osteoblast specific genes expression levels in patient and normal orbital bone. Both osteocalcin (**a**) and alkaline phosphatase (**b**) were highly expressed in patient’ orbital bone. *P < 0.05, compared with the normal

To explore the factors that may affect the upgrowth of orbit bone in Crouzon syndrome patients,we examined two osteoblast related genes expressions *OC* and *ALP*. The results clearly showed that *OC* and *ALP* was remarkably increased in the Crouzon syndrome patient compared with normal orbital bone (Fig. 3a and b). These data further highlight the clinical importance of *FGFR2* in craniofacial growth.

## Discussion

Crouzon syndrome, although of variable penetrance is accounting for 4.8% of all craniosynostoses [1]. Currently, Crouzon syndrome is commonly diagnosed when patients present with obviously facial structure abnormalities and growth patterns. However, exophthalmos was happening largely before these presentations as exophthalmos was visualized on fetal ultrasound at 35 weeks for orbital growth retardation [3]. Clinically, exophthalmos can be diagnosed in pregnancy. Furthermore, through chorionic villus biopsy, Schwartz using PCR by targeting of *FGFR2* known mutation found within the pregnant mother, successfully demonstrated diagnosis of the pregnancy Crouzon syndrome [19]. A number of mutations of *FGFR2* have been well characterized to date and a few mutations are identified affecting cell proliferation such as osteoblasts and chondrocytes in vitro [9–11]. Thus, any minor changes of *FGFR2* and the spatiotemporal planes can then inevitably lead to primordial bone changes [20].

Given the scant array of case reports in the literature, treatment of Crouzon syndrome borrows from the general craniosynostosis literature in addition to existing case reports. Ultimately, molecular genetic testing should be performed in the context of genetic counseling, and be complemented by multidisciplinary clinical follow-up by a multiple interdisciplinary teams to preventing the process of traumatic repeat surgeries for patients struck with the disease.

In summary, our results provide direct evidence that *FGFR2* mutation is associated with shallow orbits in Crouzonsyndrome that confirms the crucial and conserved role of *FGFR2* during orbit development. This finding is useful and valuable for genetic counseling and prenatal diagnosis in families with Crouzon syndrome without serious ocular disorders.

## Conclusions

Crouzon syndrome is characterized by exophthalmos and divergent strabismus to ocular hypertelorism in ophthalmology mainly because shallow orbits. Our study identified one *FGFR2* mutation (p.G338R, c.1012G > C) that increased osteogenesis gene expression from one Chinese family with 5 generation. The weak findings in this study provide little support for mutation of *FGFR2* may lead to reduce the volume of orbit in Crouzon syndrome patients. If future evidence supports this finding, it may provide reassurance to multiple interdisciplinary teams concerned about early intervention on this disorder.

Further research could explore how *FGFR2* gene can cause shallow orbits in Crouzon syndrome.

## Abbreviations

ALP: Alkaline phosphatase
Arg: Arginine
BOV: Bony orbital volume
CT: Computed tomography
FC: Figure count
Gly: Glycine
HPFL: Horizontal palpebral fissure length ()
IPFH: Vertical interpalpebral fissure height
LP: Light perception
OC: Osteocalcin
